# Gastric side effects and the stomach dosimetric analysis in left-sided breast cancer radiotherapy in free-breathing and deep inspiration breath-hold technique

**DOI:** 10.1186/s13014-021-01963-7

**Published:** 2022-01-03

**Authors:** Dong Yang, Ying Piao, Fengshun Yuan, Hongtao Chen, Ding Zhang, Xianming Li

**Affiliations:** 1 Department of Radiation Oncology, Shenzhen People’s Hospital, The First Affiliated Hospital of Southern University of Science and Technology, Dongmenbei Road 1017, Shenzhen, 518000 Guangdong People’s Republic of China; 2 Center for AIDS/STD Control and Prevention, Sichuan Center for Disease Control and Prevention, Zhongxue Road 6, Chengdu, 610051 Sichuan People’s Republic of China

**Keywords:** Gastric side effects, Left-sided breast cancer, Radiotherapy, DIBH

## Abstract

**Background:**

Adjuvant radiotherapy following surgery reduces the local recurrence and improves the prognosis. However, a considerable part of patients developed digestive reaction in daily treatment. In order to explore the correlation between breast radiotherapy and gastric toxicity, we investigated the clinic symptoms and stomach dose during DIBH or FB mode while left-sided breast cancer patients (LSBCP) receiving radiotherapy.

**Methods:**

In the study, 124 LSBCP received adjuvant radiotherapy after surgery at our department were analyzed clinical characteristics and enquired about gastrointestinal side effects after treatment. Moreover, dosimetric parameters were assessed.

**Results:**

There was no statistically significant difference between the two groups in age, T staging, N staging, hormone receptors, human epidermal receptor-2 (HER2), surgical methods, fractionated regimen, and chemotherapy conditions. However, larger stomach volumes and higher fractionated dose (Dmax/F) were associated with a statistically significantly greater risk for acute radiotherapy toxicity. In addition, the use of the DIBH gating technique (FB/DIBH) reduced the incidence of digestive reactions.

**Conclusion:**

In order to cut down gastric side effects after breast radiotherapy, large meals should be avoided before treatment. DIBH treatment should be implemented in centers where conditions are satisfied to reduce radiotherapy side effects. Furthermore, dose limitation in stomach should be considered when the radiotherapy plan was formulated, especially for the patients treated with hypofractionated radiotherapy.

## Introduction

Breast cancer is one of the most common cancers diagnosed in women [1]. Currently, breast cancer treatment is based on comprehensive therapy, consists of surgery, chemotherapy, radiotherapy, targeted therapy, and endocrine therapy [2]. Adjuvant radiotherapy following surgery reduces the local recurrence and improves the prognosis [3, 4]. But radiotherapy is associated with many side reactions in adjacent organs, including heart [5, 6], lungs [7], contralateral breast [8, 9], skin [10] and brachial plexus [11, 12]. Actually, a considerable part of patients developed digestive reaction in daily treatment, especially in those suffered left-sided breast cancer. In a study published in 2017, 64% (67/106) breast cancer patients suffered radiation-induced nausea and vomiting (RINV) during radiotherapy [13]. Digestive reaction might result in weight loss, even further cause changes in body shape, which should be rigorously avoided during radiotherapy. So it’s essential for radiation therapists to recognize the digestive symptoms during radiotherapy in left-sided breast cancer patients (LSBCP).

As gastric side effects have not been widely attracted attention to, the main objective of this study is to prove the existence of gastric side effects in the process of breast cancer radiotherapy. Possible related primary factors responsible for the detrimental effects might be found by analyzing the patients’ characteristics. With the suspected factors, radiation therapists can keep the side effects occurrence as low as possible.

As is well known, digestive symptoms are always associated with radiation treatment to the upper abdomen, such as gastric, pancreatic, and bile-duct carcinomas [14]. It has been identified the OARs in the upper abdomen as the peripheral trigger zone of emesis [15, 16]. This anatomical area contains the neural vagal connections, fibres collected in the coeliac plexus, gastroesophageal junction, and gastric mouth through which the afferent pathway of emesis to the brain stem develops. Among the upper abdomen organs, stomach possesses a relatively close distance to the left breast. The distance varies with the change of stomach volume. So it’s important to realize the relationship between the dose in stomach and the digestive symptoms in LSBCP.

Nowadays, the deep inspiration breath-hold (DIBH) technique is widely used to decrease the radiation dose to many organs, such as the heart, left anterior descending coronary artery (LAD), lungs, contralateral breast, and other organs in LSBCP [17–19]. But whether the radiation dose of stomach can be reduced in DIBH has not been noticed. To explore the correlation between the respiration control technique and gastric toxicity, we investigated the RINV and stomach dose during DIBH or FB mode while LSBCP were receiving radiotherapy.

## Methods and materials

### Patient population

From June 2020 to December 2020, a total of 124 consecutive LSBCP received adjuvant radiotherapy after surgery (with or without adjuvant chemotherapy and targeted therapy) at our department was analyzed clinical characteristics and dosimetric parameters and enquired about gastrointestinal side effects after treatment. Patient characteristics are presented in Table 1. The fractionated dose schemes of this group of patients are listed in Table 2.

**Table 1:** patient characteristics

Category	FB	DIBH
Number of patients	74	50
Median age (years)	49 (31–74)	45 (33–56)
Breast conserving surgery (yes/no)	35/39	33/17
T category		
Is	3	4
1	21	24
2	36	19
3	8	2
4	6	1
N category		
0	31	25
1	24	17
2	13	3
3	5	4
x	1	1
Hormone receptor (±)	50/24	44/6
HER-2 (±)	24/50	15/35
Hypofractionated RT/conventional RT	33/41	40/10
Chemotherapy (yes/no)	63/11	35/15

*DIBH* deep inspiration breath-hold, *FB* free-breathing, *RT* radiation therapy

**Table 2:** The fractionated dose schemes of patients

Category	Dose		FB	DIBH
	Course I	Course II		
	Whole breast (lumpectomy bed)	Lumpectomy bed		
Number of patients			74	54
Hypofractionated RT			31	40
15F ± 5F	2.7 Gy/F	2.0 Gy/F	22	26
16F ± 5F	2.7 Gy/F	2.0 Gy/F	4	7
15F	2.7 Gy/F (3.33 Gy/F)	–	5	7
Conventional RT			43	10
25F ± 5F	2.0 Gy/F	2.0 Gy/F	27	7
27F	2.0 Gy/F (2.22 Gy/F)	–	16	3

In the hypofractionated RT cohort, most patients received the treatment regimen in which whole breast irradiation was followed by photon or electron boost of 10 Gy in five fractions to the tumor bed.For the patients whose treatment course was interrupted by holiday, one fraction was added in order to ensure treatment effect (16F ± 5F). Or the whole breast and boost planning target volumes were treated simultaneously.In the conventional team, the left breast and tumor bed were treated simultaneously for the patients who underwent breast-conserving radiotherapy. While for patients without metal clips in their tumor bed, electron boost of 10 Gy in five fractions was conducted after 25 fractions of left breast treatment. The radiotherapy dose of the second course was not collected in this study

*RT* radiotherapy

### CT simulation

All patients received computer tomography (CT) (Siemens SOMATOM Definition AS) scans in the supine position lying on a vacuum mat, with both arms abducted above the head. The patients treated in DIBH mode underwent a free-breathing CT scan and a breath-hold CT scan in the same treatment position. The breath-hold CT scan was conducted using the ELEKTA Active Breathing Coordinator™ device (ELEKTA). While other patients in FB mode just underwent a free-breathing (FB) CT scan. Single-slice CT images were obtained using 3-mm thickness from the third cervical vertebrae to the 15 cm below the diaphragm, including the whole chest and stomach. CT images were transferred to the radiotherapy planning system (TPS).

### Contouring and treatment planning

Target contouring was conducted in Eclipse 13.6. Clinical target volume (CTV) was contoured conformed to the Radiation Therapy Oncology Group breast atlas (www.rtog.org). Stomach was delineated along the serosa. The heart, lungs, contralateral breast, and spinal cord were also contoured. The planning target volume (PTV) was a 5 mm isotropic expansion of the CTV but was limited to 1 mm below the skin surface. To reduce operator variability and to maintain consistency, all of the organs at risk (OAR) and target volumes were contoured by the same physician and reviewed by two senior physicians. As for the patients treated in DIBH mode, contouring was conducted on the DIBH CT.

Treatment for all the patients was planned using Elekta Monaco TPS v5.11.0 (Elekta) software. The prescription dose to the PTV was presented in Table 2. Radiotherapy was performed with 6MV X-ray using two tangential inverse planned conformal beams which directions were set to minimize the OARs dose (80% of total prescription), and two to five intensity-modulated fields (20% of total prescription). For the patients with breast-conserving surgery, an extra dose of 10 Gy (5 fractions of 2 Gy) was added for tumour bed volume in the hypofractionated radiotherapy treatment. The additional 10 Gy was achieved with one or two IMRT beams or electron boost. For the patients whose treatment course was interrupted by holiday, one fraction was added to ensure treatment effect (16F ± 5F). Or the whole breast (2.7 Gy/F) and boost (3.33 Gy/F) PTVs were treated simultaneously to shorten the treatment period. While in the conventional radiotherapy group, the whole breast and boost PTVs were treated simultaneously for the patients who underwent breast-conserving radiotherapy, except those without metal clips in their tumour bed. Electron boost of 10 Gy in five fractions was conducted after 25 fractions of left breast treatment for those patients without metal markers. As digestive symptoms often occurred during the whole breast radiotherapy, the treatment of the second course (if existed) was not taken into account in this study, probably because of the small volume of the tumour bed.

### Treatment workflow

At the first outpatient visit, patients were asked to maintain a regular living habit and regular diet. Any chemotherapeutics couldn’t be carried out during the treatment, especially oral capecitabine. Then the radiotherapy treatment was delivered at the same time of a day with the CT scan. All patients were treated with Axesse linear accelerator (Elekta) with daily set-up according to the skin markers and cone-beam CT (CBCT) image registration once a week.

After the end of the therapy, patients were asked a set of questions about toxicity information in the clinic visits. Questions usually included the incidence and severity of nausea, vomiting, bad appetite, diarrhoea, loss of weight, and other common side effects. The upper digestive toxicities were graded according to the systems proposed by RTOG.

### Statistical analysis

Student’s t test was used to compare continuous variables statistically, and chi-square and Fisher’s exact tests were used for categorical variables. Statistical analyses were performed using SPSS 23.0 (SPSS IBM Inc., Armonk, New York). A p-value < 0.05 was considered statistically significant.

## Results

**Fig. 1:**
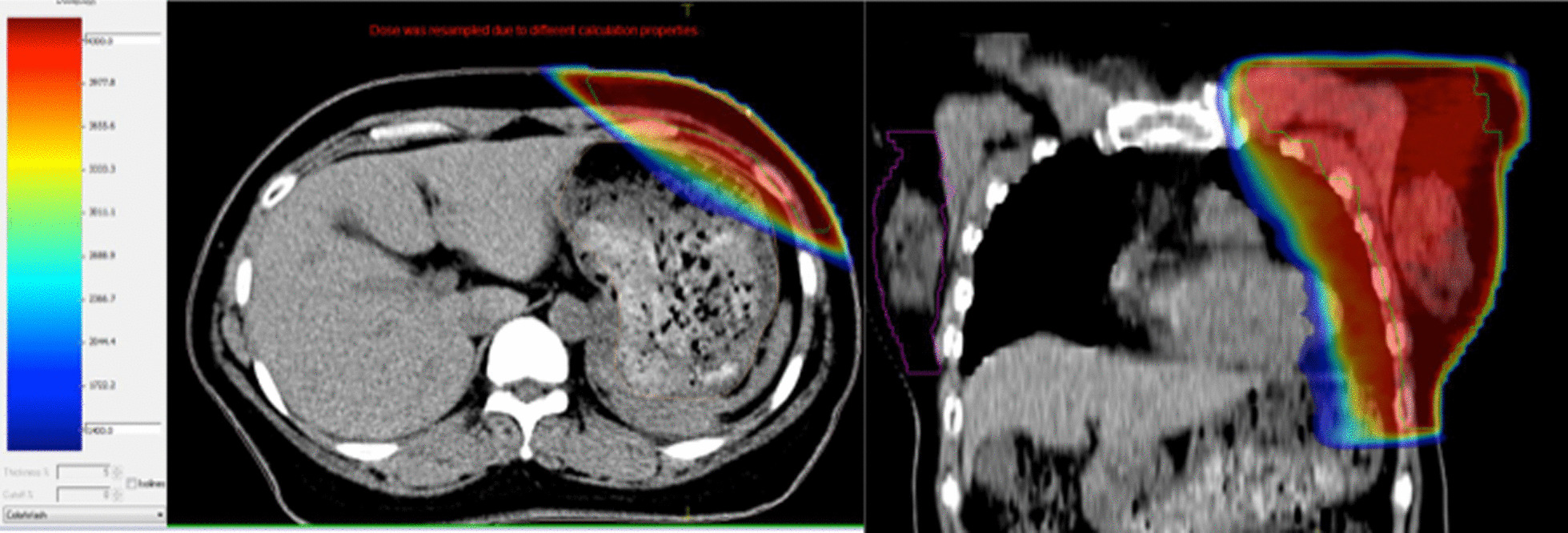
Example of a patient who suffered grade II toxicity. A substantial portion of the stomach was contained in high-dose area

In our study, a total of 124 LSBCP was included with 74 in the FB cohort and 50 in the DIBH cohort. Of all the patients, 12.1% (15/124) patients developed gastric symptoms: 11 patients suffered grade I toxicity (loss of appetite, nausea), 3 patients with grade II toxicity (loss of appetite, nausea, vomit, lose weight ≤ 5%), and 1 with grade III toxicity (loss of appetite, nausea, vomit, lose weight ≥ 5%). The isodose distribution in the stomach on simulation CT of one of the patients who suffered grade II toxicity is shown in Fig. 1.

Among the cases with or without gastric symptoms, it was found that there was no statistically significant difference between the two groups in age (P = 0.367), T staging (P = 0.682), N staging (P = 0.279), hormone receptors (P = 0.355), human epidermal receptor-2 (HER2) (P = 1.000), surgical methods (P = 0.585), fractionated regimen (P = 0.273), and chemotherapy conditions (P = 1.000). However, stomach volume (P = 0.047) and the FB mode (FB/DIBH) (P = 0.026) were associated with a statistically significantly greater risk for acute radiation toxicity (see Table 3). What’s more, higher fractionated dose in stomach was found statistically significant associated with the gastric toxicity, including Dmax/F (D1cc/F, dose delivered to a 1cm^3^ volume of the stomach/fraction) (P < 0.001), D60cc/F (dose delivered to a 60cm^3^ volume/fraction) (P = 0.001), D30cc/F (P = 0.001), and D10cc/F (P < 0.001).

**Table 3:** Analysis of characteristics of gastric complications in breast cancer patients [n(%)]

	Gastric side effects		χ^2^-value/t value	P-value
	No (%)	Yes (%)		
Age			0.91	0.367
	47.5 ± 8.1	49.6 ± 10.8		
T stage			1.50	0.682
T1	48 (88.9)	6 (11.1)		
T2	47 (87.0)	7 (13.0)		
T3	8 (80.0)	2 (20.0)		
T4	6 (100.0)	0 (0.0)		
N stage			1.35	0.279
N0	48 (84.2)	9 (15.8)		
N1, N2, N3, Nx	61 (91.0)	6 (9.0)		
Hormone receptor (±)			0.78	0.355
Negative	25 (83.3)	5 (16.7)		
Positive	84 (89.4)	10 (10.6)		
HER2			0.03	1.000
Negative	75 (88.2)	10 (11.8)		
Positive	34 (87.2)	5 (12.8)		
Breast conserving surgery (yes/no)			0.46	0.585
No	48 (85.7)	8 (14.3)		
Yes	61 (89.7)	7 (10.3)		
Fractionated regimen			1.47	0.273
Conventional radiotherapy	47 (92.2)	4 (7.8)		
Hypofractionated radiotherapy	62 (84.9)	11 (15.1)		
Chemotherapy (yes/no)			0.01	1.000
No	23 (88.5)	3 (11.5)		
Yes	86 (87.8)	12 (12.2)		
Stomach volume (m^3^)			2.01	0.047
	371.6 ± 149.1	458.5 ± 209.4		
Use of respiratory gating technique			5.17	0.026
DIBH	48 (96.0)	2 (4.0)		
FB	61 (82.4)	13 (17.6)		
Dmax/F			9.44	0.000
	122.5 ± 89.8	241.8 ± 35.8		
D10cc/F			6.34	0.000
	63.3 ± 63.4	174.1 ± 63.6		
D30cc/F			4.27	0.001
	34.4 ± 38.8	114.2 ± 71.0		
D60cc/F			3.98	0.001
	18.7 ± 20.2	69.8 ± 49.2		

**Fig. 2:**
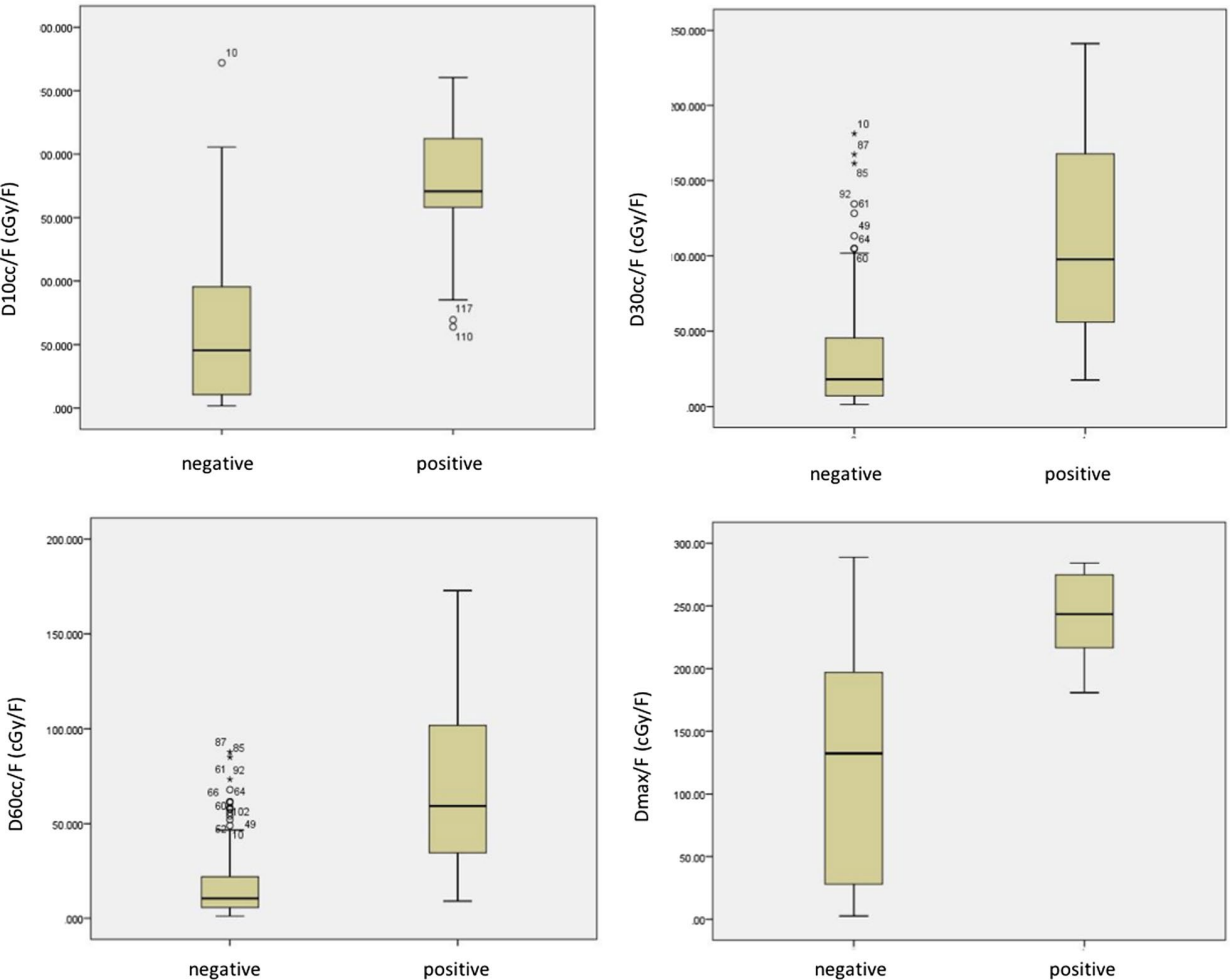
The stomach D60cc/F, D30cc/F, D10cc/F, and Dmax/F was significantly lower in the negative symptoms cohort

The stomach irradiation dose and volume were associated with digestive reactions. As is shown in Table 3, the stomach Dmax/F was 122.5 ± 89.8 Gy/F in the negative symptoms cohort vs. 241.8 ± 35.8 Gy/F in the positive cohort (P < 0.001). Similar results can be observed in the stomach D10cc/F (63.3 ± 63.4 vs 174.1 ± 63.6 Gy/F), D30cc (34.4 ± 34.8 vs 114.2 ± 71.0 Gy/F), and D60cc/F (18.7 ± 20.2 vs 69.8 ± 49.2 Gy/F). Statistically significant reductions in the stomach dose of the negative cohort were observed in all of the four dosimetric parameters (see Fig. 2).

**Fig. 3:**
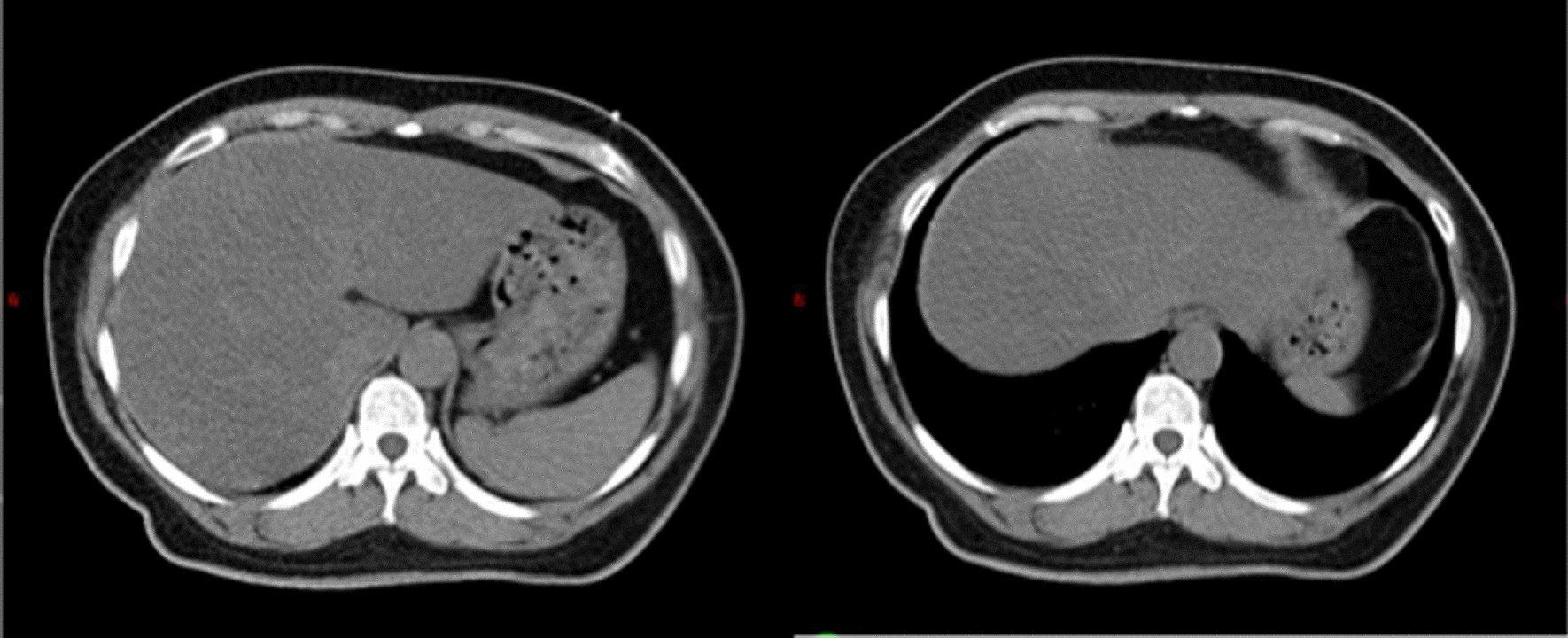
Computed tomography scans for both FB (left) and DIBH (right) at the same axial location in 1 patient. During DIBH, the stomach was pushed downwards and backwards by the left lung, leading to lower dose distribution

Consistent with clinical experience, 2/50 (4.0%) patients suffered gastric symptoms received radiotherapy in DIBH mode and 13/74 (17.6%) in the FB mode, with a statistically significant difference (P = 0.026). The different locations of the stomach during FB and DIBH can be presented in Fig. 3.

**Fig. 4:**
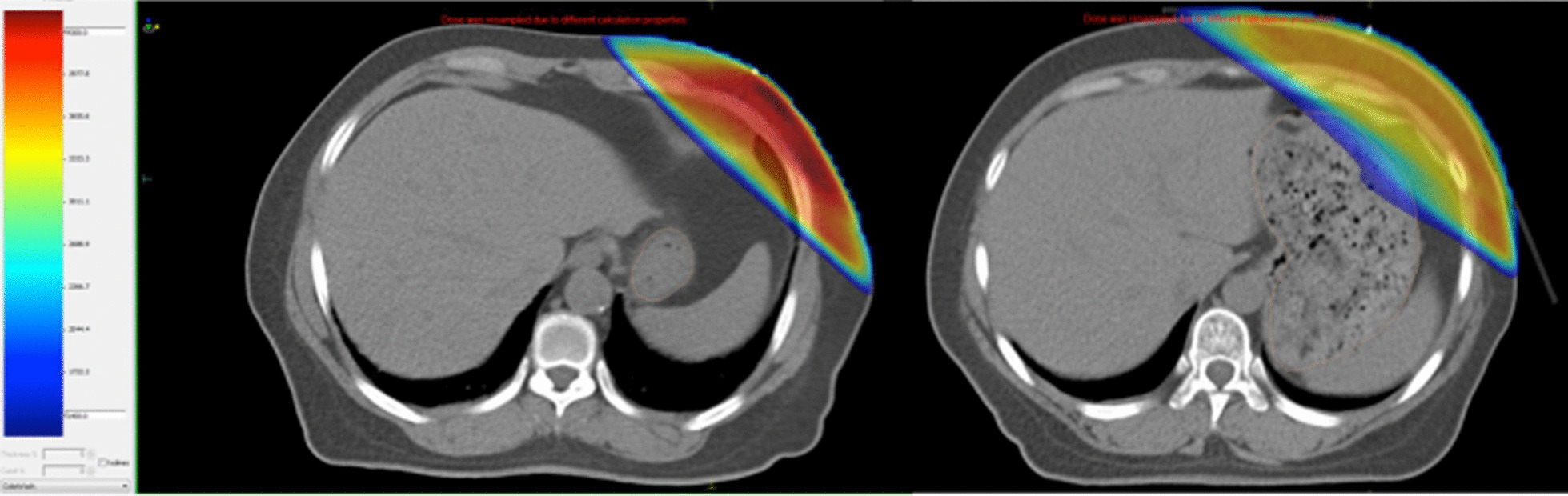
Computed tomography scans for 2 patients with stomachs in small size (left) and huge size (right). The dosimetric distributed in stomach quite different on account of different stomach volumes

In addition, correlation can be demonstrated between stomach volumes and digestive reactions. A reduction in the stomach volume was seen in the patients with negative symptoms, from 458.5 ± 209.4 to 371.6 ± 149.1 m^3^, with a significant difference (P = 0.047). The relationship between the stomach volume and the dose distribution can be depicted vividly in Fig. 4.

## Discussion

This article reports a crucial experience, justifying that radiotherapy of the LSBCP led to an early gastric reaction, such as nausea, vomit, and loss of weight. It is well established that radiotherapy-induced gastric reaction is usually associated with irradiation for upper abdomen tumours, such as pancreatic, gastric, or liver cancer [14]. Total body irradiation can also cause vomiting. As we mentioned earlier, RINV usually resulted from the irradiation of OARs in the upper abdomen as the peripheral trigger zone of emesis. Because of the deep abdominal location, the OARs are normally excluded from high radiation doses in breast radiotherapy. This is the reason why the RINV in breast cancer had been neglected for such a long time. For most patients, stomach dose can be significantly reduced if adequate blocking is used. But for the patients who have huge a stomach, the large volume of the stomach squeezes the organs around, including the left lung upward, leading to a relatively shorter distance between stomach and left breast. As a result, in our clinical practice, a considerable section of patients was found to endure grade I-II gastrointestinal toxicity ever since the hyper-fractionated radiotherapy plan was conducted in our centre. RINV usually occurred immediately or several hours after breast radiotherapy. At the end of radiation, some patients had weight loss more or less.

In our study, acute radiation-related gastric complications were associated with the irradiation dose of stomach. With a higher Dmax/F, D60cc/F, D30cc/F, or D10cc/F, the incidence of gastric complications grew significantly. As is shown in Fig. 1, most of the patients who suffered from gastric poisoning had a considerable portion of the stomach in the high-dose zone. A higher max dose or a more extensive high-dose zone in stomach is usually correlated with a larger stomach volume, as is depicted in Fig. 4. The dosimetric distribution in the stomach is quite different on account of varying stomach volumes. A larger stomach volume leads to the closer distance between the stomach wall and the chest wall, and then shortens the gap between the stomach and the PTV.

Numerous publications have investigated the dosimetric advantages in heart, LAD, lung, and liver for breast cancer radiotherapy in DIBH mode [17–23]. However, this is the first report that observed the stomach dose in DIBH and FB. Ever since the DIBH technique was applied in our centre, a considerable portion of LSBCP received radiotherapy in the new respiratory gating mode. There was a significant difference in the occurrence rate of gastric toxicity between the FB and DIBH groups. Variability in the stomach dose can be due to physiological factors: during DIBH, the lungs become enlarged and push the diaphragm downward, leading to a relatively long distance between stomach and PTV and lower radiation dose compared with FB. Therefore, in DIBH mode, advantageous dose distribution in the stomach can be achieved, resulting in a lower probability of gastric symptoms.

In addition, although there was no relationship between the gastric side effects and fractionated regimen in this study, fewer fractions always lead to higher PTV single dose irradiation. The prescription dose to PTV was 2.0 Gy ~ 2.22 Gy/F in the conventional fraction cohort, while in hypofraction mode the dose was 2.7 Gy ~ 3.33 Gy/F. Because of the short distance between left breast and stomach in certain situations, higher dose fractionated irradiation in PTV could result in more irradiation dose/F in the stomach. However, a considerable portion of the patients (40/50) was treated in hypofraction mode under the DIBH technique, the occurrence of gastric symptoms rate might be decreased because of the stomach movement.

As the application of the hypofractionated regimen becomes more widespread, more and more LSBCP might experience upper digestive tract side reactions. Poor appetite, nausea, and vomiting during radiotherapy could result in weight loss in patients, which will lead to changes in body shape. As a result, the safety and accuracy of treatment could be cut down, especially for the PTV located in body surfaces, such as in the breast and chest walls. In Mary Feng’s study focused on intrahepatic malignancies radiotherapy, gastric bleeds might occur after radiotherapy at a median time of 4 months [24]. In another study published in 2009, the risk of gastric cancer as a second malignant tumour rose with the increasing stomach mean dose (Dmean) [25]. Therefore, the gastric dose in left breast radiotherapy should be attached importance to.

Emami et al. [26] estimated doses with a 5% risk at 5 years (TD5/5) for late stomach toxicities in 1991. The TD5/5 estimated for severe gastric complications after the whole-stomach irradiation dose of 50 Gy, and 1/3 volume of the whole-organ irradiation dose of 60 Gy, had been widely accepted as a dose limit guideline. However, the dose limit was established in the era of three-dimensional conformal radiation therapy (3D-CRT). As new techniques, such as intensity-modulated radiation therapy (IMRT), volumetric modulated arc therapy (VMAT), tomotherapy, and even intensity-modulated proton therapy (IMPT) [27–29], had been widely implemented in breast cancer radiotherapy, new dose limitation of stomach should be recommended. There were no gastrointestinal symptom references to suggest a safe dose with the current radiotherapy techniques, a reduction to gastric symptoms should be conducted by proposing a new stomach dose limitation when the radiotherapy plan was formulated. Considering that most centres usually couldn’t scan the entire stomach in breast cancer simulation, we collected Dmax, D60cc, D30cc and D10cc as statistic variables. The current work demonstrated that Dmax/F, D60cc/F, D30cc/F and D10cc/F in stomach were associated with gastric side effects. So further study is required to validate a specific OAR limitation achievable for lower stomach side effects.

When appraising our products, both advantages and limitations should be taken into account in the meantime. Up to now, seldom evidence published in the literature demonstrated that radiotherapy of LSBCP is correlated with an increased risk of gastric reaction. In the current study, we showed an increased incidence rate in LSBCP received hyper-fractionated radiotherapy. What’s more, we suggested dose limitations that radiation oncologists can use to check their radiotherapy plan. Meanwhile, there are several limitations to the study. First, the stomach wall may change depending on the volume of the contents. Planned doses for 3D-CRT + IMRT cannot be flexibly modified or adjusted according to day-to-day or real-time organ displacements around the stomach, resulting in different digestive symptoms after everyday treatment. Second, seldom publications studied the stomach dose limitation as an OAR in breast cancer, so there weren’t enough applicable references. Third, gastroscopy was not conducted after radiotherapy to investigate acute radiation-related gastric toxicity. Moreover, real long-term side effects were not reported because of the short follow-up period.

In conclusion, a huge stomach could be closer to the breast PTV, so large meals should be avoided before treatment. DIBH treatment should be implemented in centres where conditions are satisfied to reduce radiotherapy side effects. Furthermore, dose limitation in stomach should be considered when the radiotherapy plan was formulated, especially for the patients treated with hypofractionated radiotherapy.

## Data Availability

The datasets used and/or analysed during the current study are available from the corresponding author on reasonable request.
